# Stochastic Thermodynamics of an Electromagnetic Energy Harvester

**DOI:** 10.3390/e24091222

**Published:** 2022-08-31

**Authors:** Luigi Costanzo, Alessandro Lo Schiavo, Alessandro Sarracino, Massimo Vitelli

**Affiliations:** Department of Engineering, University of Campania “Luigi Vanvitelli”, 81031 Aversa, Italy

**Keywords:** electromagnetic energy harvester, stochastic thermodynamics, work fluctuations, correlation functions

## Abstract

We study the power extracted by an electromagnetic energy harvester driven by broadband vibrations. We describe the system with a linear model, featuring an underdamped stochastic differential equation for an effective mass in a harmonic potential, coupled electromechanically with the current in the circuit. We compare the characteristic curve (power vs. load resistance) obtained in experiments for several values of the vibration amplitude with the analytical results computed from the model. Then, we focus on a more refined analysis, taking into account the temporal correlations of the current signal and the fluctuations of the extracted power over finite times. We find a very good agreement between the analytical predictions and the experimental data, showing that the linear model with effective parameters can describe the real system, even at the fine level of fluctuations. Our results could be useful in the framework of stochastic thermodynamics applied to energy harvesting systems.

## 1. Introduction

Harvesting energy from environmental noise is a fundamental task in many contexts, from the microscales, as in Brownian motors driven by thermal fluctuations [1,2,3,4], to the macroscopic world, as for random vibrations in ships, bridges, railroad cars, and aircraft [5]. In order to treat systems where fluctuations play a central role, in recent years, thermodynamics concepts such as heat, work and entropy have been extended to the realm of stochastic processes and non-equilibrium systems, paving the way to the development of stochastic thermodynamics [6,7]. The interest in the study of these fluctuating quantities, such as heat and work defined along a single stochastic trajectory of the system, is motivated by the search for optimization protocols in models of engines where fluctuations are dominant [8] and by the general symmetry properties that the distribution functions have been shown to satisfy, both theoretically [9,10,11] and experimentally [12,13].

From a more practical perspective, in Internet of Things applications, energy harvesting systems, converting ambient energy into electricity, can represent an alternative solution to disposable batteries [14,15]. That is why energy harvesters have found application in very different fields [16,17,18]; see also [19,20]. In particular, vibration energy harvesters, which convert the wide available mechanical energy of vibrations into electric energy, have proven particularly attractive [21,22]. These devices are mainly based on piezoelectric materials or electromagnetic induction [23,24]. Both piezoelectric and electromagnetic vibration harvesters are typically operated in resonant structures and can efficiently operate only near resonance, even if electromagnetic harvesters are characterized by higher powers with respect to piezoelectric ones. In the literature, the study of vibration harvesters forced by non-sinusoidal or random vibrations has been deeply carried out in case of piezoelectric technology [25,26,27], and in case of hybrid electromagnetic-piezoelectric systems [28,29]. On the other hand, electromagnetic vibration harvesters are typically studied under purely sinusoidal vibrations tuned to their resonance frequency. Analysis of electromagnetic harvesters in the presence of random vibrations can be found in [30,31], but the literature lacks a stochastic analysis of the fluctuations and distributions of their relevant quantities.

In this work, we consider a linear model for an electromagnetic energy harvester driven by white noise, amenable for analytical solution, and compare in detail the theoretical predictions from the model with real experiments. The model consists of an underdamped Langevin equation describing the stochastic motion of an effective mass in the presence of a harmonic potential and electromechanically coupled with an external variable, the current in the circuit, whose dynamics is not directly affected by the noise. The model is, by construction, out of equilibrium, since detailed balance is broken by the coupling with the current variable. This can also be interpreted as a feedback mechanism acting on the mass and introducing a memory effect in the system [32,33]; see also [27] where we performed a similar analysis on a piezoelectric energy harvester. We first fit the effective model parameters to the characteristic curve of the system, namely the extracted power versus the load resistance, and then analyze the time correlation functions of the current and the fluctuations of the extracted work over finite time intervals. We find that the linear model with effective parameters can reproduce very well both the time decay of the correlation function and the work fluctuations at different times.

## 2. Experimental Setup

**Figure 1:**
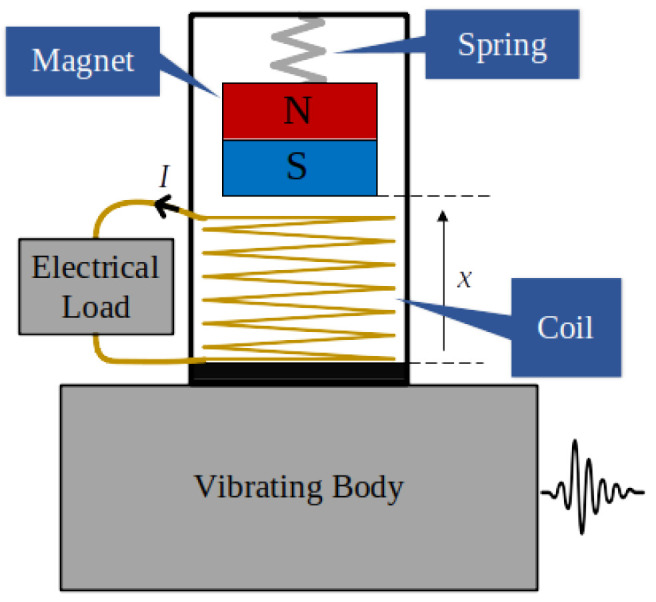
Schematic representation of an electromagnetic vibration energy harvester.

A typical electromagnetic vibration energy harvester, schematically shown in Figure 1, is based on a permanent magnet, which is attached to the housing by means of a spring system and can move relative to a coil fixed to the housing when a vibration is applied. The coil is connected to an electrical load, and the relative displacement between the magnet and the coil leads to the conversion of the mechanical energy of vibrations into electrical energy. The maximization of the energetic performance can be carried out on the basis of mechanical tuning techniques, acting on the mechanical characteristics, or on the basis of maximum power point tracking techniques, acting on power electronic converters connected to the coil terminals [34,35,36,37].

**Figure 2:**
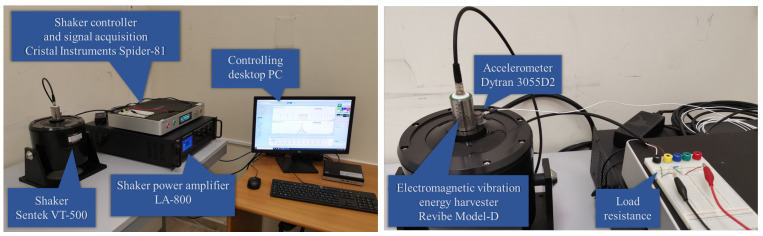
(**Left** panel) Experimental setup employed for the tests; (**Right** panel) zoom of the harvester, the accelerometer and the load resistance.

The experimental setup that has been employed in all the tests is shown in Figure 2. In particular, the considered harvester is the Model-D by Revibe. It is an electromagnetic vibration energy harvester characterized by a mechanical resonance frequency of about 100 Hz. The harvester is placed on a shaker, the VT-500 by Sentek, used as the source of desired vibrations. In order to generate the shaker vibrations, its driving current is provided by an LA-800 power amplifier. The Spider-81 controller by Crystal Instruments implements a closed loop vibration control by providing the driving signal to the power amplifier on the basis of the acceleration of the shaker vibrations measured by the Dytran 3055D2 accelerometer. It is worth noting that, as shown in Figure 2 (right), the considered electromagnetic harvester comes as a black box that, apart from the forcing by the input vibration, is accessible only at the electric terminals. Therefore, even if its detailed mechanical structure cannot be accessed, it will be shown that a linear model with effective parameters can well reproduce the measured dynamics.

**Figure 3:**
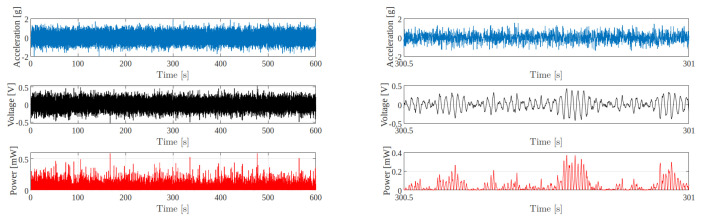
(**Left** panel) Waveforms of the input acceleration, of the voltage and the power across the load resistance (for R=490 Ω) recorded during the experimental test with a Gaussian noise input signal with a standard deviation 0.5 g. (**Right** panel) Zoom on a small time window of 0.5 s.

The harvester Revibe Model-D was forced by broadband vibrations of Gaussian type with a sampling rate f=5 kHz and different standard deviations (0.25 g, 0.5 g and 0.75 g), which were generated with MATLAB software and provided to the shaker controller. In Figure 3, the waveforms of the input acceleration, of the voltage and of the power across the load resistance recorded during one of the experimental tests are shown as an example.

## 3. Theoretical Model

In order to reproduce the dynamics observed in the experimental systems, we consider the following underdamped linear Langevin equation

(1) $$\begin{array}{ccc} \dot{x} & = & v \end{array}$$

(2) $$\begin{array}{ccc} M\dot{v} & = & -k_{s}x-\gamma v-\theta I+M\xi \end{array}$$

(3) $$\begin{array}{ccc} L_{c}\dot{I} & = & \theta v-(R_{c}+R)I, \end{array}$$

where ξ is white noise with zero mean and variance $\langle \xi \left(t\right)\xi \left(t^{\prime }\right)\rangle =2D_{0}\delta \left(t-t^{\prime }\right)$. In the above equations, *x* represents the position of the magnet with respect to the coil, *v* represents its velocity, *M* represents the mass, γ represents the viscous damping due to the air friction, $k_{s}$ is the elastic constant of the spring effective system, *I* is the current at the electrical terminals, $L_{c}$ and $R_{c}$ are the coil inductance and resistance, respectively, *R* is the load resistance and θ is the effective electromechanical coupling factor. In particular, during its movement, the permanent magnet, apart from the viscous damping force of the medium and the elastic force of the spring, is subject to the electromagnetic (Lorentz) force due to the interaction between the current *I* flowing into the coil and the induction field of the magnet. As shown in detail in [38], θ takes into account the coil geometrical properties, its number of turns and the magnetic field strength. It is the coefficient that, in the linear model expressed by Equation (2), links the electromagnetic force and the current at the electrical terminals. This current is due to the electromotive force induced in the coil that, as shown in Equation (3), is proportional to the magnet speed through θ.

In our experimental setup, noise is introduced through the vibrations applied by the shaker to the energy harvester. The role of the shaker mimics the effect of real broad-band vibrations that can be measured for instance in a car or in an aircraft. In our model, such fluctuations are treated as white noise, affecting the dynamical equation for the mass velocity.

The linear model can be solved (see below and the Appendix A for details) to obtain the mean extracted power as a function of the parameters

(4) $$P_{harv}=\langle I^{2}\rangle R=\frac{D_{0}\theta^{2}M^{2}R}{\left[\gamma \left(R+R_{c}\right)+\theta^{2}\right]\left[\gamma L_{c}+M\left(R+R_{c}\right)\right]+\gamma k_{s}L_{c}^{2}},$$

where 〈…〉 represents an average over the stationary state.

### Fitting Parameters

**Figure 4:**
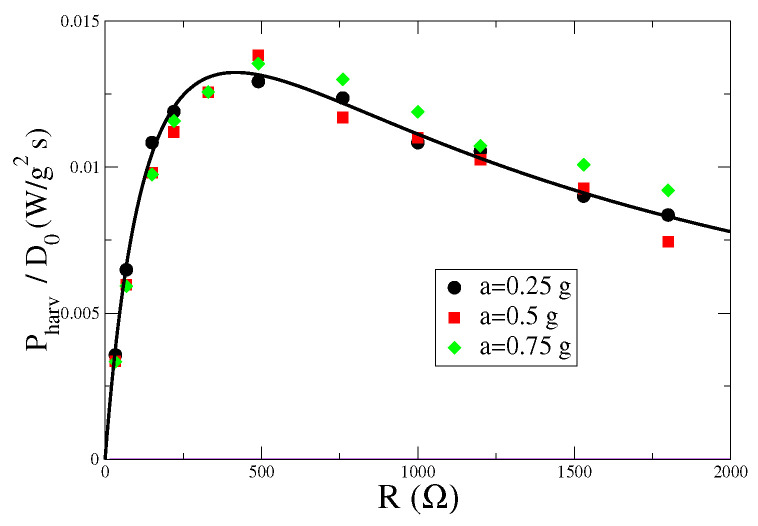
$P_{harv}$ (rescaled by $D_{0}=a^{2}\Delta t/2$, where Δt=1/f) as a function of the load resistance *R*, for different values of the input acceleration *a*, measured in units of gravity acceleration *g*. Symbols are experimental data, while the line corresponds to the best fit obtained from Formula (4).

Using Equation (4), we can fit the parameters of the linear model to the experimental data. Because of the large number of parameters, we also exploit the temporal decay of the correlation function of the current signal (see next section) in order to better estimate some of them (in particular the effective viscosity γ). We also impose a constraint on the parameters $k_{s}$ and *M*, such that $k_{s}={\left(2\pi *100\right)}^{2}M$, dictated by the mechanical resonance of the harvester. We obtain the following values: γ=1.80±0.05 Kg/s, M=0.048±0.005 Kg, $k_{s}=18811\pm 50$ Kg/s${}^{2}$, θ=29.9±0.5 N/A, $R_{c}=227.6\pm 0.5$ Ω, $L_{c}=0.124\pm 0.005$ H, which give the analytical curve reported in Figure 4.

## 4. Temporal Autocorrelation Function

Beyond the study of the mean extracted power, we are interested in exploring the possibility that the model could also well reproduce other features observed in the experimental system. Therefore, in this section, we focus on the autocorrelation function of the measured current I(t), namely the quantity $C_{II}\left(t\right)=\langle I\left(t\right)I\left(0\right)\rangle$ in the stationary state. This provides information on the characteristic time-scales in the system.

An analytical expression for $C_{II}\left(t\right)$ can be obtained explicitly due to the linear nature of the model. In particular, we introduce the column vector $X={\left(x,v,I\right)}^{T}$ and define the coupling matrix

(5) $$A=\left(\begin{array}{ccc} 0 & -1 & 0\\ \frac{k_{s}}{M} & \frac{c}{M} & \frac{\theta }{M}\\ 0 & -\frac{\theta }{L_{c}} & \frac{R_{c}+R}{L_{c}} \end{array}\right),$$

so that the Equations (1)–(3) can be rewritten in vectorial form as

(6) $$\dot{X}=-AX+\eta ,$$

where $\eta ={\left(0,\xi ,0\right)}^{T}$. Then, defining the covariance matrix $\sigma =\langle X^{T}X\rangle$ as

(7) $$\sigma =\left(\begin{array}{ccc} \sigma_{xx} & \sigma_{xv} & \sigma_{xI}\\ \sigma_{vx} & \sigma_{vv} & \sigma_{vI}\\ \sigma_{Ix} & \sigma_{Iv} & \sigma_{II} \end{array}\right),$$

at stationary, one has the constraint [39]

(8) $$D=\frac{A\sigma +\sigma A^{T}}{2},$$

where *D* is the noise matrix

(9) $$D=\left(\begin{array}{ccc} 0 & 0 & 0\\ 0 & D_{0} & 0\\ 0 & 0 & 0 \end{array}\right).$$

The solution of Equation (8) provides the covariance matrix as a function of the model parameters (see Appendix A). In order to compute the correlation function $C_{II}\left(t\right)$, we first introduce the response matrix

(10) $$G\left(t\right)=e^{-At}.$$

Then, the correlation $C_{II}\left(t\right)$ can be expressed in terms of the matrix elements of G(t) and of the covariance matrix as follows [39]:

(11) $$C_{II}\left(t\right)=G_{Ix}\left(t\right)\sigma_{xI}+G_{Iv}\left(t\right)\sigma_{vI}+G_{II}\left(t\right)\sigma_{II},$$

where the symbols $G_{ij}\left(t\right)$ denote the response of the variable i∈{x,v,I} to a perturbation on the variable j∈{x,v,I}. This algebraic computations have been handled with Mathematica. The general form of $C_{II}\left(t\right)$ is an exponential decay, modulated by sinusoidal oscillations.

**Figure 5:**
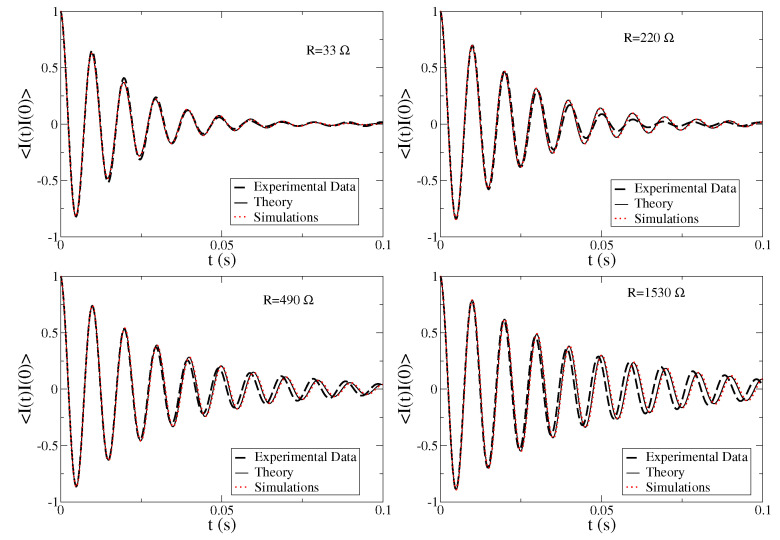
Autocorrelation function of the current signal $C_{II}\left(t\right)=\langle I\left(t\right)I\left(0\right)\rangle$ measured in the experiments (dashed black line), theory (thin black line) and numerical simulations (red dots) for different values of the load resistance *R* at driving acceleration a=0.25 g.

The autocorrelation from the experimental data is obtained by averaging the whole recorded temporal signal (excluding initial and final transient regimes) on a sliding window of 0.1 s. We then compare the results from experiments with the correlations obtained analytically and with numerical simulations of the model Equations (1)–(3), as reported in Figure 5, for several values of the load resistance *R* and for acceleration a=0.25 g (other values of *a* show the same behaviors). First, we observe that analytical predictions and numerical simulations perfectly agree, confirming the correctness of the analytical approach. More interestingly, the comparison with experimental data shows that the model very well reproduces the characteristic frequency of the signal, and the amplitude decay of the oscillations, with small discrepancies for larger values of the load resistance *R*.

## 5. Stochastic Energetics

We now analyze the system from a stochastic thermodynamics [7] approach. In particular, we focus on the work fluctuations at finite times, comparing the model predictions obtained from numerical simulations, with experimental data. According to the prescription of stochastic energetics [40], we define the heat exchanged along a trajectory in a time interval [0,τ] with the surrounding medium as

(12) $$Q_{ex}\left(\tau \right)=-\int_{0}^{\tau }\gamma v{\left(t\right)}^{2}dt,$$

and the energy fed into the system from the external driving as the integral of the injected power $P_{inj}=M\xi \left(t\right)v\left(t\right)$

(13) $$E_{inj}\left(\tau \right)=M\int_{0}^{\tau }\xi \left(t\right)v\left(t\right)dt,$$

where the product is meant according to the Stratonovich definition. Here, we consider these two contributions separately, at variance with standard Brownian systems where the two terms arise from the coupling of the particle with the fluid. Indeed, in our system, thermal fluctuations are completely negligible, whereas $Q_{ex}$ is the heat dissipated to the environment due to the viscosity γ. The injected power is instead due to the white noise introduced by the (macroscopic) shaker.

Next, using the Langevin Equation (2), we can rewrite these two terms as follows

(14) $$\begin{array}{ccc} Q_{ex}\left(\tau \right)+E_{inj}\left(\tau \right) & = & \int_{0}^{\tau }\left[-\gamma v{\left(t\right)}^{2}+Mv\left(t\right)\xi \left(t\right)\right]dt\\ & = & \frac{1}{2}M\left[v{\left(\tau \right)}^{2}-v{\left(0\right)}^{2}\right]+\frac{1}{2}k_{s}\left[x{\left(\tau \right)}^{2}-x{\left(0\right)}^{2}\right]+\theta \int_{0}^{\tau }v\left(t\right)I\left(t\right)dt\\ & = & \Delta E+W^{\prime }\left(\tau \right), \end{array}$$

where

(15) $$\Delta E=\frac{1}{2}M\left[v{\left(\tau \right)}^{2}-v{\left(0\right)}^{2}\right]+\frac{1}{2}k_{s}\left[x{\left(\tau \right)}^{2}-x{\left(0\right)}^{2}\right]$$

is the mechanical energy variation, and

(16) $$W^{\prime }\left(\tau \right)=\theta \int_{0}^{\tau }v\left(t\right)I\left(t\right)dt$$

can be interpreted as the electromechanical work, which allows us to read Equation (14) as the first law for stochastic thermodynamic quantities. The effective work extracted by the harvester is defined as the difference between $W^{\prime }\left(\tau \right)$ and the heat dissipated in the parasite resistance $R_{C}$

(17) $$W\left(\tau \right)=W^{\prime }\left(\tau \right)-R_{C}\int_{0}^{\tau }I{\left(t\right)}^{2}dt.$$

Next, exploiting Equation (3), the work *W* can be related to the dissipated heat in the load resistance *R* in the time interval [0,τ]

(18) $$Q_{diss}\left(\tau \right)=R\int_{0}^{\tau }I{\left(t\right)}^{2}dt,$$

via the relation

(19) $$W\left(\tau \right)=Q_{diss}\left(\tau \right)+\frac{L_{c}}{2}\left[I{\left(\tau \right)}^{2}-I{\left(0\right)}^{2}\right].$$

The difference between the two quantities is given by a term non-extensive in time, that can be neglected for long trajectories.

In our experimental setup, we have direct access to the current *I* in the load circuit and, thus, to the work *W*, according to Equations (18) and (19). Therefore, we can compare the behavior of the fluctuations of these quantities with those obtained from the numerical simulations of the linear model, where the parameters have been fixed through the fitting procedure described above. Let us note that here, the extracted power is directly related to the dissipated heat by the Joule effect in the load resistance. Therefore, by definition, since it involves a square of the electrical current, the work is always positive. This rules out the possibility of observing negative fluctuations, even at small times, and thus the validity of a work fluctuation relation cannot be explored.

**Figure 6:**
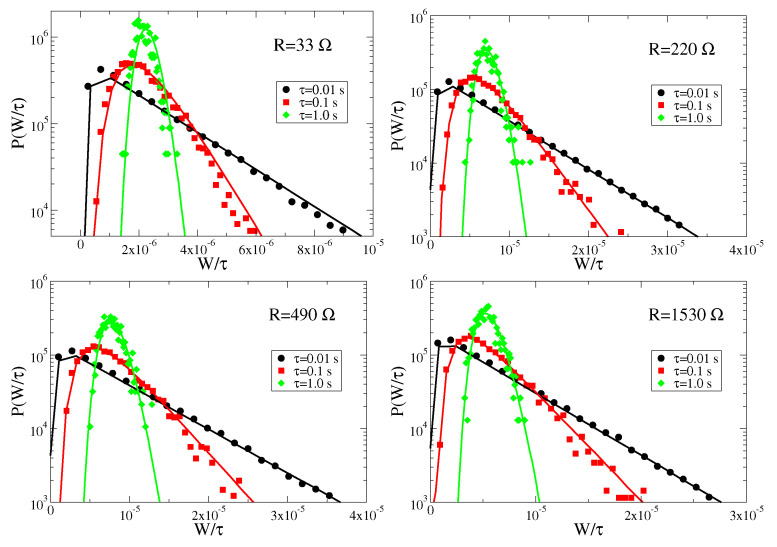
Probability distribution of the work W in a time interval τ for different values of the load resistance *R* at driving acceleration a=0.25 g. Symbols represent experimental data, whereas lines represent numerical simulations.

In Figure 6 we compare the power fluctuations measured in the experiments for different times and several parameters, with the results of numerical simulations of the linear model. A very good agreement is found in all cases. This shows that the effective linear model provides an accurate description of the real system, not only for average values, but even at the fine level of fluctuations.

Let us also comment on the functional forms of the work distributions at finite times. We observe that, before converging towards a Gaussian distribution, the probability density function shows very long tails at large values of W/τ. This means that, over small time intervals, there is a finite probability to extract a work much larger than the average value even if the most probable value is smaller than the average.

Finally, we observe that the efficiency of the system can be defined as the ratio $\eta =P_{harv}/\langle P_{inj}\rangle$, where, from Equation (13), $\langle P_{inj}\rangle =MD_{0}$. Due to the linearity of the model, the dependence on $D_{0}$ appears as a prefactor in both the expressions and thus cancels out in the ratio. This results in a behavior of η as a function of the load resistance *R* similar to the one reported in Figure 4, with a peak around R=500Ω. As a function of the other parameters, as expected, we find that the efficiency increases with the electromechanical coupling θ and decreases with the parasite resistance $R_{c}$.

## 6. Conclusions

We have shown that a linear model of an underdamped Langevin equation with an electromechanical coupling can very well reproduce the behavior of a real electromagnetic energy harvester, not only with respect to the average values of the measured current, but also with respect to its temporal correlations and fluctuations. The analysis of these quantities and the comparison between analytical prediction and experimental data allow one to obtain a better estimate of the effective parameters for the model.

The study of the stochastic energetics of the system could be extended to other relevant quantities, such as entropy production. In order to address this issue from an experimental perspective, however, a more complete access to the dynamical variables is necessary. In particular, this requires the measurements of the position and velocity of the mass, which are not directly accessible with the actual experimental setup. Indeed, for instance, as illustrated in Refs. [32,33] for a similar linear model, the contribution of the medium entropy production takes the form $\Delta s_{m}=-1/T\int_{0}^{\tau }dt\left[M\dot{v}\left(t\right)+\theta \tilde{I}\left(x,v\right)+k_{s}x\left(t\right)\right]v\left(t\right)$, where *T* is related to the noise amplitude $D_{0}$ and $\tilde{I}\left(x,v\right)$ is obtained from a projection over the current distribution probability [33] and explicitly involves the variables *x* and *v*. We plan to address the study of entropy production in future works, in particular for a piezoelectric energy harvester [27], where direct access to all the dynamical variables is possible, and therefore, an experimental measurement of entropy production can be compared to theory and simulations.

## Data Availability

Experimental and numerical data are available on request from the authors.

## Appendix A

The solution of the linear system of Equation (8) provides the elements of the covariance matrix

(A1) $$\begin{array}{ccc} \sigma_{xx} & = & \frac{D_{0}M^{2}\left(\left(R+R_{c}\right)\left(\gamma L_{c}+M\left(R+R_{c}\right)\right)+k_{s}L_{c}^{2}\right)}{\gamma k_{s}\left(\left(R+R_{c}\right)\left(\gamma L_{c}+M\left(R+R_{c}\right)\right)+k_{s}L_{c}^{2}\right)+\theta^{2}k_{s}\left(\gamma L_{c}+M\left(R+R_{c}\right)\right)}\\ \sigma_{xv} & = & \sigma_{vx}=0\\ \sigma_{xI} & = & \frac{D_{0}\theta L_{c}M^{2}}{\gamma \left(\left(R+R_{c}\right)\left(\gamma L_{c}+M\left(R+R_{c}\right)\right)+k_{s}L_{c}^{2}\right)+\theta^{2}\left(\gamma L_{c}+M\left(R+R_{c}\right)\right)}\\ \sigma_{vv} & = & \frac{D_{0}M\left(\gamma L_{c}\left(R+R_{c}\right)+k_{s}L_{c}^{2}+\theta^{2}L_{c}+M{\left(R+R_{c}\right)}^{2}\right)}{\gamma \left(\left(R+R_{c}\right)\left(\gamma L_{c}+M\left(R+R_{c}\right)\right)+k_{s}L_{c}^{2}\right)+\theta^{2}\left(\gamma L_{c}+M\left(R+R_{c}\right)\right)}\\ \sigma_{vI} & = & \frac{D_{0}\theta M^{2}\left(R+R_{c}\right)}{\gamma \left(\left(R+R_{c}\right)\left(\gamma L_{c}+M\left(R+R_{c}\right)\right)+k_{s}L_{c}^{2}\right)+\theta^{2}\left(\gamma L_{c}+M\left(R+R_{c}\right)\right)}\\ \sigma_{Ix} & = & \frac{D_{0}\theta L_{c}M^{2}}{\gamma \left(\left(R+R_{c}\right)\left(\gamma L_{c}+M\left(R+R_{c}\right)\right)+k_{s}L_{c}^{2}\right)+\theta^{2}\left(\gamma L_{c}+M\left(R+R_{c}\right)\right)}\\ \sigma_{Iv} & = & \frac{D_{0}\theta M^{2}\left(R+R_{c}\right)}{\gamma \left(\left(R+R_{c}\right)\left(\gamma L_{c}+M\left(R+R_{c}\right)\right)+k_{s}L_{c}^{2}\right)+\theta^{2}\left(\gamma L_{c}+M\left(R+R_{c}\right)\right)}\\ \sigma_{II} & = & \frac{D_{0}\theta^{2}M^{2}}{\gamma \left(\left(R+R_{c}\right)\left(\gamma L_{c}+M\left(R+R_{c}\right)\right)+k_{s}L_{c}^{2}\right)+\theta^{2}\left(\gamma L_{c}+M\left(R+R_{c}\right)\right)}. \end{array}$$

From the last term $\sigma_{II}=\langle I^{2}\rangle$, we get Equation (4).
